# Hypovirulence-associated mycovirus epidemics cause pathogenicity degeneration of *Beauveria bassiana* in the field

**DOI:** 10.1186/s12985-023-02217-6

**Published:** 2023-11-03

**Authors:** Zhengkun Zhang, Wenbo Guo, Yang Lu, Qin Kang, Li Sui, Hongyu Liu, Yu Zhao, Xiaowei Zou, Qiyun Li

**Affiliations:** 1 Institute of Plant Protection, Jilin Academy of Agricultural Sciences, Jilin Key Laboratory of Agricultural Microbiology, Key Laboratory of Integrated Pest Management on Crops in Northeast China, Ministry of Agriculture and Rural Areas, Changchun, 130033 People’s Republic of China; 2 College of Plant Protection, Jilin Agricultural University, Changchun, 130118 People’s Republic of China; 3 Institute of Zoology, Chinese Academy of Sciences, Beijing, 101408 People’s Republic of China; 4 Jilin Agricultural Science and Technology University, Jilin, 132109 People’s Republic of China

**Keywords:** Mycovirus, Epidemic, Entomogenous fungus, *Beauveria bassiana*, BbCV2, Biological control, Hypovirulence mechanism

## Abstract

**Background:**

The entomogenous fungus *Beauveria bassiana* is used as a biological insecticide worldwide, wild *B. bassiana* strains with high pathogenicity in the field play an important role in controlling insect pests via not only screening of highly virulent strains but also natural infection, but the pathogenicity degeneration of wild strains severely affected aforementioned effects. Previous studies have showed that multiple factors contributed to this phenomenon. It has been extensively proved that the mycovirus infection caused hypovirulence of phytopathogenic fungi, which has been used for plant disease biocontrol. However, it remains unknown whether the mycovirus epidemics is a key factor causing hypovirulence of *B. bassiana* naturally in the field.

**Methods:**

Wild strains of *B. bassiana* were collected from different geographic locations in Jilin Province, China, to clarify the epidemic and diversity of the mycoviruses. A mycovirus *Beauveria bassiana* chrysovirus 2 (BbCV2) we have previously identified was employed to clarify its impact on the pathogenicity of host fungi *B. bassiana* against the larvae of insect pest *Ostrinia furnacalis*. The serological analysis was conducted by preparing polyclonal antibody against a BbCV2 coat protein, to determine whether it can dissociate outside the host fungal cells and subsequently infect new hosts. Transcriptome analysis was used to reveal the interactions between viruses and hosts.

**Results:**

We surprisingly found that the mycovirus BbCV2 was prevalent in the field as a core virus in wild *B. bassiana* strains, without obvious genetic differentiation, this virus possessed efficient and stable horizontal and vertical transmission capabilities. The serological results showed that the virus could not only replicate within but also dissociate outside the host cells, and the purified virions could infect *B. bassiana* by co-incubation. The virus infection causes *B. bassiana* hypovirulence. Transcriptome analysis revealed decreased expression of genes related to insect epidermis penetration, hypha growth and toxin metabolism in *B. bassiana* caused by mycovirus infection.

**Conclusion:**

*Beauveria bassiana* infected by hypovirulence-associated mycovirus can spread the virus to new host strains after infecting insects, and cause the virus epidemics in the field. The findings confirmed that mycovirus infection may be an important factor affecting the pathogenicity degradation of *B. bassiana* in the field.

**Supplementary Information:**

The online version contains supplementary material available at 10.1186/s12985-023-02217-6.

## Background

Mycoviruses can infect phytopathogenic fungi, reducing their pathogenicity toward plants, and providing a means to achieve biological control of plant disease [1]. For example, hypovirulent strains of *Cryphonectria parasitica* infected with mycovirus Cryphonectria hypovirus 1 (CHV1) were released in forest, the expansion and spread of chestnut blight were effectively controlled by its epidemic [2]. *Sclerotinia sclerotiorum* hypovirulence-associated DNA virus 1 (SsHADV-1) can directly infect hyphae of the host phytopathogenic fungus *Sclerotinia sclerotiorum * in vitro with high efficiency, and direct spraying of the virion on plant leaves can control Sclerotinia disease [3]. Similarly, *Sclerotinia sclerotiorum* partitivirus 1 (SsPV1) can inhibit the growth of *S. sclerotiorum* and significantly decrease host pathogenicity [4], and the mycovirus Heterobasidion partitivirus 13 strain an1 (HetPV13-an1) can diminish the pathogenicity and growth rate of host fungi [5].

*Beauveria bassiana* is an entomogenous fungus that has been used worldwide and developed into a variety of commercial agents for biological control of pest insects in forestry and agriculture [6–8]. However, virulence toward insect pests decreases during the preservation and application of *B. bassiana* strains, limiting the efficient application of waste resources [9]. The mechanism of its hypovirulence has been extensively studied, including host specialisation and genetic diversity [10, 11], the impact of environmental factors [9, 12], nutritional factors [13, 14], morphology and physicochemical characteristics of plant surfaces where fungi are applied [15, 16]. Moreover, insects have pathogen recognition systems and immune defence capabilities that can also counteract entomogenous fungi [17–19].

In recent years, more and more mycoviruses have been isolated and characterised from *B. bassiana* [20–25], most of which do not impact the virulence of *B. bassiana*. However, a few mycoviruses possess the ability to alter the biological characteristics and virulence of *B. bassiana*. Kotta and Coutts (2017) found that 16 of 75 strains of *B. bassiana* from different locations around the world contained mycoviruses, among which mycoviruses *Beauveria bassiana* polymycovirus 1 (BbPmV-1) and *Beauveria bassiana* polymycovirus 3 (BbPmV-3) were found to affect pigment deposition, spore production and colony growth of their host fungus by interfering with basic metabolic pathways [26, 27]. However, it is unclear whether mycoviruses have the ability to cause epidemics, and thereby induce hypovirulence of *B. bassiana* populations in the field, even though mycoviruses are able to undergo interspecific, intraspecific and vertical transmission in *Beauveria* spp. [28]. Therefore, whether mycoviruses are transmitted through direct hyphal contact or dissociate outside to infect other host fungi following their replication in host fungal cells remains to be elucidated.

To explore these questions, we selected the mycovirus *Beauveria bassiana* chrysovirus 2 (BbCV2) that have identified in previous work [29], which infects various *Beauveria* species [28], and investigated whether the virus (1) is prevalent and genetically stable in wild *B. bassiana* populations in the field; (2) can dissociate outside host fungus cells to infect other strains; and (3) can decrease the pathogenicity of the host *B. bassiana* strain; we also (4) probed the mechanism through which mycoviruses affect host fungi. The results help to reveal whether mycoviruses are key factors causing *B. bassiana* hypovirulence in preservation and field applications.

## Materials and methods

### Detection of mycoviruses in wild strains of *B. bassiana*

In the autumn of 2020, we collected and identified 106 strains of *B. bassiana* isolated from *Ostrinia furnacatis* Guenee muscardine cadavers in corn fields in seven different locations in Jilin Province, China (Additional file 1: Table S1). All strains were stored at −80 °C. The tested strains were activated and cultured on PDA medium. Ten strains from each collection site were randomly selected, and the aforementioned methods were used for dsRNA extraction and RT-PCR detection to determine the BbCV2 infection status [29].

### Genetic diversity analysis

Three strains of *B. bassiana* were randomly selected from each different collection site confirmed to be infected by virus BbCV2 by dsRNA extraction and RT-PCR. Three pairs of primers (Additional file 2: Table S2) were designed for full-length amplification of nucleotide sequences of the RNA-dependent RNA polymerase (RdRp) gene of virus BbCV2 (GenBank no. MW314841.1). Products obtained by RT-PCR amplification were extracted from 1% agarose electrophoresis gels, cloned using a TA/Blank Zero Cloning Kit (Vazyme, Nanjing, China), transformed into competent *Escherichia coli* DH5-α cells (Vazyme) by heat shock, and sequenced by Sangong Bioengineering Co. Ltd. (Shanghai, China). The complete sequence of the BbCV2 RdRp gene was obtained by splicing using DNAstar7.1 (DNASTAR, Inc., Madison, USA) and DNAMAN version 9 (Lynnon Biosoft, Vaudreuil, Canada). Modification and splicing of the measured cDNA gene sequence were assessed using Snap Gene 6.0.2 (Dotmatics Ltd, Windhill, UK) and DNAMAN (LynnonBiosoft, Vaudreuil, Canada) 9. MEGA11 (Mega Limited, Auckland, New Zealand) and DNAsp5 (University of Barcelona, Barcelona, Spain) were used to construct a phylogenetic tree of virus BbCV2 in the selected *B. bassiana* strains collected from different locations in Jilin Province, and the genetic distance was calculated.

### Antibodies preparation and viruses-specific detection

The dsRNAs of two mycoviruses BbCV2 and a previously reported polymycovirus BbPmV-4 [28] were extracted from host fungus, and the cDNA of coat protein genes (*BbCV2-CP* and *BbPmV-4-CP*) of the two viruses were amplified by PCR using specific primers possessing suitable restriction enzyme sites. Construction of recombinant vector, genetic transformation and protein expression were performed as previously described [32]. Recombinant CP proteins were purified from inclusion bodies using Ni NTA affinity chromatography (SOLARBIO, Beijing, China), and were subjected to sodium dodecyl sulfate polyacrylamide gel electrophoresis (SDS-PAGE) analysis.

Animal immunisation for polyclonal antibody preparation was performed as described previously [32] using six large-eared Japanese rabbits (Liaoning Changsheng Biotechnology Co. Ltd. Shenyang, China). The titre of polyclonal antibody detection was determined using indirect enzyme linked immunosorbent assay (ELISA) as previously described [33, 34]. OD_450_ values of polyclonal antibodies (P) and of negative serum wells (N) were determined. Antibody titre tests were conducted three times and average values of P and N were calculated at each dilution. An average P/N ration > 2 was considered positive.

Virus-infected and virus-free strains of *B. bassiana* were cultured on PDA for 10 days, fungal hyphae was scraped for total protein extraction using a Fungal Protein Extraction Kit (SOLARBIO) according to the manufacturer’s instructions, and protein lysis solution was assessed by western blotting as described previously [35].

### Viruses detection within and outside *B. bassiana* cells

Five randomly selected strains of *B. bassiana* harbouring virus BbCV2 and BbPmV-4 verified by RT-PCR were used for viruses detection in vitro and in vivo by indirect ELISA. Five randomly selected viruses-free *B. bassiana* strains verified by RT-PCR served as negative controls, purified CP proteinsof the viruses served as positive control, and double-distilled water served as blank control. Fungal strains were cultured in SDY medium until hyphae formation, the supernatant and pellet were separated by centrifugation, the sediment was washed three times with sterile water to remove culture medium residues, frozen in liquid nitrogen and thawed three times, and lysed by Scientz JY98-IIIDN ultrasonic instrument (Ningbo, Zhejiang, China). Samples were centrifuged, the supernatant was removed, and the pellet was resuspended in phosphate-buffered saline (PBS). These samples and the liquid culture medium used for fungi cultivation were subjected to virus detection by indirect ELISA.

### Detection of virus BbCV2 in insect bodies

Second instar larvae of *O. furnacatis* were infected with conidia of virus-infected strain BbOFDHCV1 and virus-free strain BbOFDH1. Larvae without *B. bassiana* infection served as blank controls. Ten insects were used in each treatment, and mycelia of strain BbOFDHCV1 cultured in liquid medium served as positive controls. Four days after inoculation, insect bodies were dissected with a sterilised scalpel to obtain epidermis, which was ground in PBS for indirect ELISA detection. Additionally, longitudinally cut insect epidermis from each treatment was mixed with 500 μL 4% paraformaldehyde, fixed for 30 min, centrifuged at 4000 rpm for 15 min to remove paraformaldehyde, rinsed three times in PBS, and blocked with 5% skimmed milk at 37 °C for 1 h. Primary antibody against BbCV2-CP was diluted 1:5000, incubated with samples at 37 °C for 1 h, and secondary antibody, FITC-labelled goat anti-rabbit IgG (Thermo Fisher Scientific, Shanghai, China) at a 1:6000 dilution was incubated at 37 °C in the dark for 1 h. Samples were observed using a Leica DM RBE laser scanning confocal microscope (Zeiss, Oberkochen, Germany) at a wavelength of 488 nm for hyphae of *B. bassiana* and 495 nm for hyphae of *B. bassiana* and virions of BbCV2.

### Virus purification from mycelia and infection in vitro

To extract viral particles, *B. bassiana* strain BbOFZK152 was grown on PDA plate for ten days, the mycelia were harvested and ground to powder in the presence of liquid nitrogen. Virus purification was performed according to description of Chiba et al. (2009) [36]. Carefully extracted sucrose of different gradients and transferred to an ultracentrifugation tube, filled tube with 0.05 M sodium phosphate buffer, centrifuged at 30,000 rpm at 4 °C for 3 h, poured out the supernatant, and added 200 μL of 0.05 M sodium phosphate buffer to mix well. Indirect ELISA was used to detect viral particles in sucrose at different gradients and double stranded RNA extraction validation was performed. Mixed 5 µL of phosphate buffer containing virus with 10 mL of *B. bassiana* strain BbOFDH1-5-GFP blastspores (10^8^ spores/mL), and incubated in a constant temperature shaker with 120 rpm at 26 °C for 5 days, and centrifuged to collect the mycelium, cleaned and centrifuged for three times with sterile water to ensure the absence of virus particles. The double stranded RNA extraction and RT-PCR were employed for virus validation. The *B. bassiana* strain BbOFDH1-5-GFP blastspores (10^8^ spores/mL) cultured individually was used as control.

### Horizontal transmission of viruses BbCV2

*Beauveria bassiana* strain BbOFZK152 is naturally infected by mycovirus BbCV-2 alone [29], while BbOFDH1-5-GFP is a virus-free isogenic strain isolated from *Ostrinia furnacalis* and labelled with the phosphinothricin resistance gene *bar* and green fluorescence protein (GFP), preserved in the China General Microbiological Culture Collection Center (CGMCC No. 15673). The two strains were cultured on potato dextrose agar (PDA) at 26 °C, and stored on PDA slants at 4 °C in the Jilin Key Laboratory of Agricultural Microbiology. Isogenic strains with and without BbCV2 infection were obtained via the insect coinfection method as described previously [28]. Coinfected insect bodies harbouring the virus-infected donor strain BbOFZK152 and virus-free recipient strain BbOFDH1-5-GFP were fed at 25 °C for 7 to 10 days as described previously [30] to obtain muscardine cadavers. Conidia from fungus-infected muscardine cadavers were inoculated on Czapek-Dox Agar medium containing 200 μg/mL phosphinothricin for screening of BbOFDH1-5-GFP strains, and monoclones of 50 of these were incubated on PDA medium for 10 days to obtain mycelia for virus detection. The presence of the virus in BbOFDH1-5-GFP strains was confirmed by double strand RNA (dsRNA) extraction [31] and Reverse Transcription-polymerase Chain Reaction (RT-PCR) with primers CVR (5′-TCCGTAGGTGAACCTGCGG-3′) and CVF (5′-TCCTCCGCTTATTGATATGC-3′) specific to BbCV2. Three virus-infected BbOFDH1-5-GFP strains, designated BbOFDHCV1, BbOFDHCV2 and BbOFDHCV3, as well as three virus-free strains BbOFDH1, BbOFDH2 and BbOFDH3 derived from monospores of BbOFDH1-5-GFP, were randomly selected for virulence evaluation, virus detection and transcriptomic analysis. Evaluation of vertical spread efficiency of BbCV2 was performed as described previously [28], in which the three virus-infected strains above were subcultured on PDA medium for three generations and verified by dsRNA extraction and RT-PCR. Tests for each strain were repeated three times.

### Virulence assays

Fungal virulence toward *O. furnacalis* was determined using the dip method. Third-instar *O. furnacalis* larvae were dipped into 2 mL suspensions (10^7^ spores/mL) for 20 s and reared normally on artificial feed. *O. furnacalis* larvae were divided into seven groups (BbOFDH1, BbOFDH2, BbOFDH3, BbOFDHCV1, BbOFDHCV2 and BbOFDHCV3, and sterile 0.05% Tween-80 as a blank control). Each group included three replicates with 20 larvae in each replicate. The number of dead insects was recorded every 24 h, beginning on day 2 and ending on day 8, and survival curves was plotted using GraphPad Prism 8 (Dotmatics Ltd, Windhill, UK).

A 1 mL volume of conidia (1 × 10^7^ conidia/mL) of *B. bassiana* strains with and without virus infection were added in the same volume (50 mL) of Sabouraud Dextrose Medium with Yeast Extract (SDY) for shaking cultivation at 26 °C for 72 h, then centrifuged at 12,000 rpm for 10 min. The supernatant was removed, dried, and fungal bodies were placed in a drying oven at 40 °C for 2 days. The dry weight of all samples was measured using a 1/10,000 balance (OLABO, Jinan, China). Each fungal strain included six replicates.

### RNA-Seq data processing and analysis

Based on the results of virulence against pests, virus-infected strains BbOFDHCV1, BbOFDHCV2 and BbOFDHCV3, as well as virus-free strains BbOFDH1, BbOFDH2 and BbOFDH3 were subjected to transcriptome sequencing. Following incubation at 26 °C for 10 days, total RNA was extracted, and RNA samples were purified and used to construct six libraries. Libraries were sequenced on an Illumina HiSeq 6000 platform at Novogene Bioinformatics Technology Co. Ltd. (Beijing, China). Raw data (raw reads) were filtered and checked for sequencing error rate and GC content distribution to obtain clean reads for subsequent analysis. The genome of *B. bassiana ARSEF 2860* (NCBI accession number: ADAH00000000) was employed as the reference genome. Clean reads from each library were searched against the *B. bassiana* genome database using the HISAT2 program [37]. Gene expression levels were quantified and corrected for sequencing depth and gene length using fragments per kilobase of exon per million mapped fragments (FPKM) [38]. Differential expression analysis between groups was performed using theDESeq2 R package (1.20.0)[39] and *p*-values were adjusted to control the false discovery rate (FDR) [40]. Differentially expressed genes (DEGs) were assigned when an absolute value of log_2_ Ratio (fold change) > 1 at a threshold q-value < 0.05 was obtained (5% FDR). Gene function enrichment analysis is used to categorise the functions of genes using different databases. Gene Ontology (GO) and Kyoto Encyclopedia of Genes and Genomes (KEGG) pathways enrichment analyses of DEGs were performed using the clusterProfiler R package (3.8.1). Significantly enriched functional categories were assigned when the corrected *p*-value was less than the threshold of 0.05 [41].

### Statistical analysis

One-way analysis of variance (ANOVA) was performed using SPSS software version 26.0 (SPSS Inc., Chicago, IL, USA). Significant differences were determined with Duncan’s multiple range test.

## Results

### Epidemics and genetic diversity of BbCV2

**Fig. 1:**
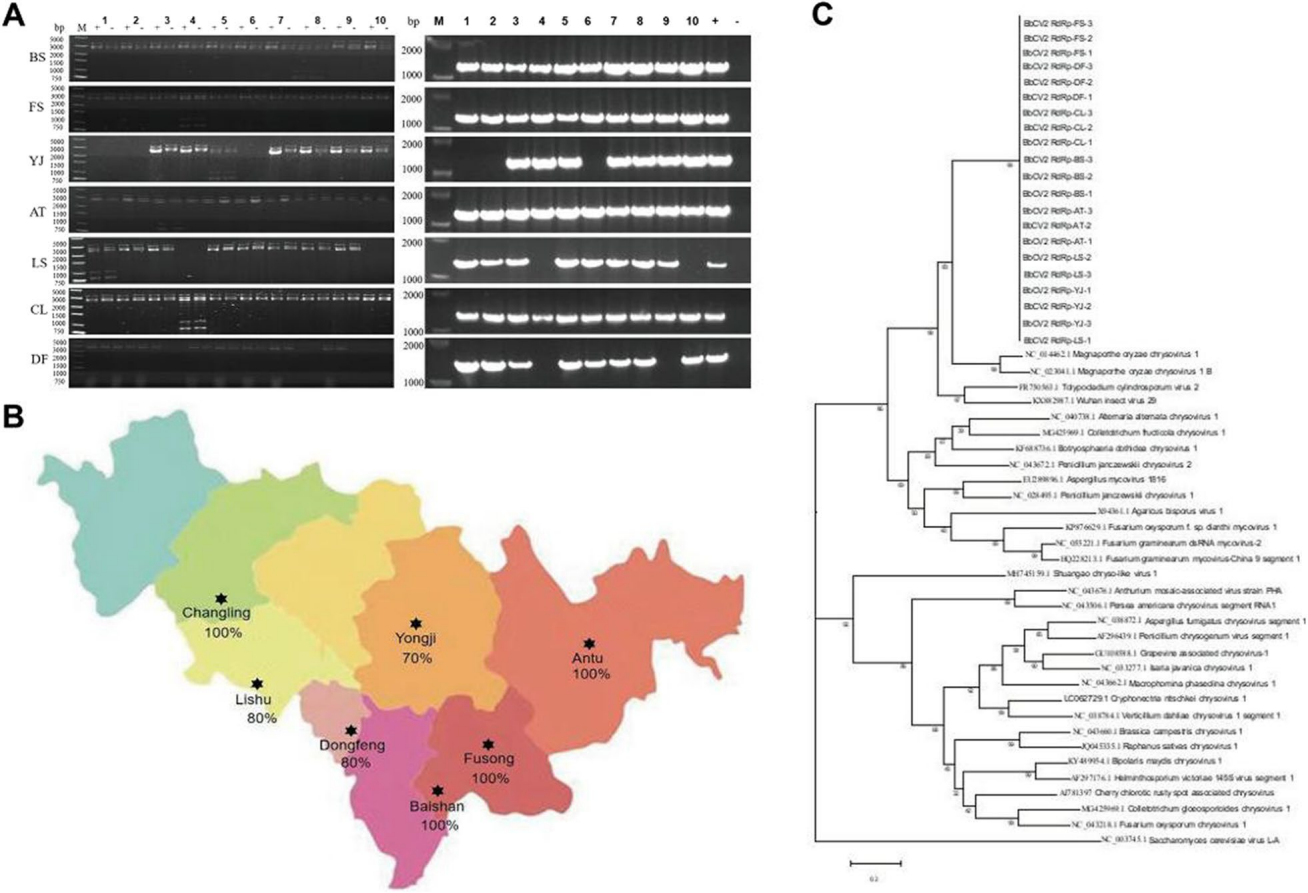
Detection and infection proportion of BbCV2 virus in *B. bassiana* strains, and analysis of genetic diversity from different collection sites. **A** dsRNA mycovirus detection of *B. bassiana* strains. Left: dsRNA extraction of all tested fungal strains; (+) = dsRNA from strains without DNase I and S1 nuclease treatment; (−) = dsRNA from strains with DNase I and S1 nuclease treatment. Right: detection of BbCV2 infection in *B. bassiana* strains by RT-PCR; (+) = original virus-infected *B. bassiana* strain of BbOFZK152 as positive control; (−) = original virus-free *B. bassiana* strain of BbOFDH-bar as negative control. **B** Proportion of BbCV2 infection in *B. bassiana* strains from different collection sites. **C** Nucleotide phylogenetic tree of virus BbCV2 collected from different regions and 32 mycoviruses

BbCV2 virus showed an extremely high virus-harbouring rate in wild *B. bassiana* strains. The results of dsRNA extraction morphology showed that *B. bassiana* strains from all different geographical sites were infected by different dsRNA viruses, and RT-PCR detection showed that BbCV2 was the major virus (Fig. 1A). The statistical results showed that the lowest infection proportion of virus BbCV2 in *B. bassiana* strains was 80% from Yongji, compared with up to 100% from Baishan, Fusong, Antu and Changling (Fig. 1B), and the average virus-harbouring rate of all tested strains was 90%. BbCV2 virus was genetically stable independent of geographical location.

Nucleotide sequences of the BbCV2 RdRp gene from 21 strains from different sites were concatenated and corrected, resulting in a total length of 3345 bp. The (+ G) model of Tamura Nei (1993) was used to evaluate the base composition of the full-length RdRp gene sequences of all BbCV2 viruses from different collection sites (Additional file 3: Table S3). The average A, U, C, and T content of RdRp gene fragments of BbCV2 viruses was 21.60%, 20.08%, 25.37% and 32.93%, respectively. The G + C content (58.31%) was higher than the A + U content (41.69%). There was no significant difference in the base content of RdRp sequences of BbCV2 viruses among different collection sites. Multiple comparison results showed that the nucleotide sequences of the RdRp gene of BbCV2 viruses in Jilin Province shared very high nucleotide sequence similarity of 99.78% and amino acid sequence similarity of up to 99.98%. Phylogenetic tree construction showed that the nucleotide sequence of the RdRp gene of 21 selected BbCV2 viruses formed an evolutionary cluster with 32 RdRp mycovirus sequences (Fig. 1C and Additional file 4: Table S4), and there was no significant differentiation phenomenon, hence the BbCV2 virus was genetically stable during epidemics in the field.

### Horizontal transmission of BbCV2

**Fig. 2:**
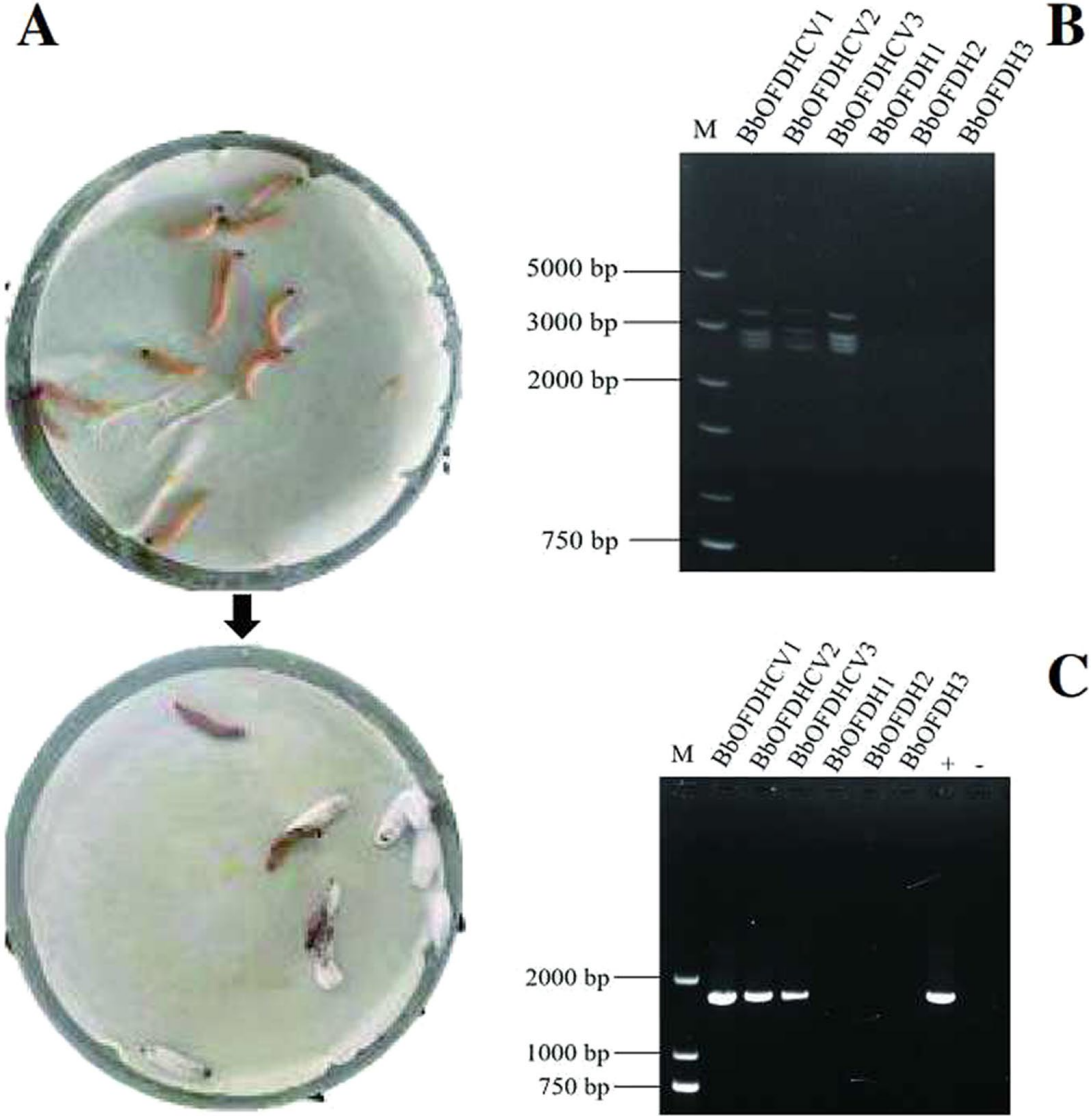
Horizontal transmission of BbCV2 via insect coinfection of *B. bassiana* strains. **A** Muscardine cadavers of insects resulting from coinfection with virus-free and virus-infected strains; **B** Detection of viral dsRNA in recipient strains via treatment with DNase I and S1 nuclease; **C** RT-PCR amplification of BbCV2 in recipient strains. Reverse transcription products from strain BbOFZK152 served as positive controls (+). Reverse transcription products were replaced by distilled deionised water in negative controls (−)

BbCV2 viruses were successfully transmitted from virus-infected strain BbOFZK152 to virus-free strain BbOFDH1-5-GFP by co-infection assay. After 10 days of coinfection, conidia from muscardine cadavers were isolated and cultured on Czapek-Dox Agar medium containing phosphinothricin, and monocolonies were cultured on PDA medium for virus infection detection. Viral dsRNA extraction and RT-PCR amplification results showed that 39 of 50 BbOFDH1-5-GFP isolates were positive. Three virus-infected strains (BbOFDHCV1, BbOFDHCV2 and BbOFDHCV3) and three randomly selected virus-free strains (BbOFDH1, BbOFDH2 and BbOFDH3) were used for subsequent experiments (Fig. 2). The results revealed stable and efficient vertical transmission of BbCV2 in *B. bassiana*. After two generations of subculture, in each generation of strains BbOFDHCV1, BbOFDHCV2 and BbOFDHCV3, the virus-harbouring rate was > 73.33 ± 5.77%, revealing no significant differences between different generations of each strain or the same generation among the three strains (Additional file 5: Table S5).

### Polyclonal antibodies production of the mycoviruses

The high-purity recombinant proteins of the two viruses BbPmV-4 and BbCV2 were obtained (Additional file 6: Fig. S1). Two highly efficient and specific polyclonal antibodies of the two viruses were successfully prepared. The titres of two purified polyclonal antibodiesagainst BbCV2 and BbPmV-4 were 1:8,192,000 and 1:256,000, respectively (Additional file 7: Fig. S2).

**Fig. 3:**
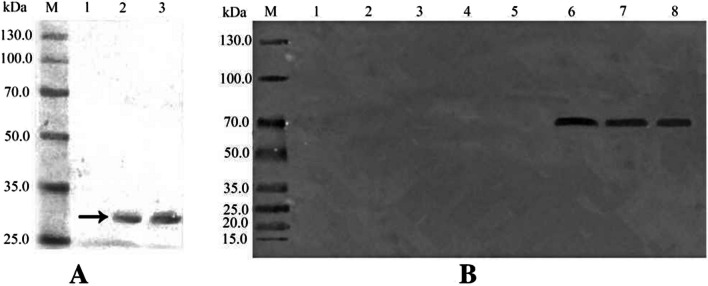
Detection of viruses in *B. bassiana* by Western blot **A** Western blotting assay of recombinant protein BbPmV-4-CP. Lane M, protein markers; Lane 1, Blank control;Lane 2–3, BbPmV-4-CP. **B** Western blotting assay of recombinant protein BbPmV-4-CP. Lane M, protein markers; Lane 1–2, blank controls; Lane 3–5, virus-free BbOFDH1-3 strains; Lane 6–8, virus-infected BbOFDHCV1-3 strains

Western blotting was performed on virus-infected and virus-free *B. bassiana* strains. Clear specific bands of 28.6 kDa (Fig. 3A) and 85 kDa (Fig. 3B) were observed for virus-infected strains but not virus-free fungal strains, indicating that the prepared antibodies could be used for viruses detection in host fungi.

### Viruses secreted outside host fungal cells

The prepared polyclonal antibodies against the two viruses BbCV2 and BbPmV-4 were used to detect the presence of the viruses both in and outside fungal cells infected by the two viruses via indirect ELISA. The results showed that both fungal hyphae and liquid medium samples were positive (P/N value > 2; Additional file 8: Fig. S3A and B), and the viruses concentration in the supernatant was significantly higher than that in the mycelium. Furthermore, identical results were observed for all insect epidermis samples infected with the BbCV2 infected fungal strain, for which the *p*-value was more than twice that of the negative control and the group infected with the virus-free fungal strain, confirming the presence of the virus BbCV2 in insect bodies (Additional file 8: Fig. S3C). The two viruses displayed the ability to dissociate outside host fungi cells not only in liquid culture media but also in insect bodies.

**Fig. 4:**
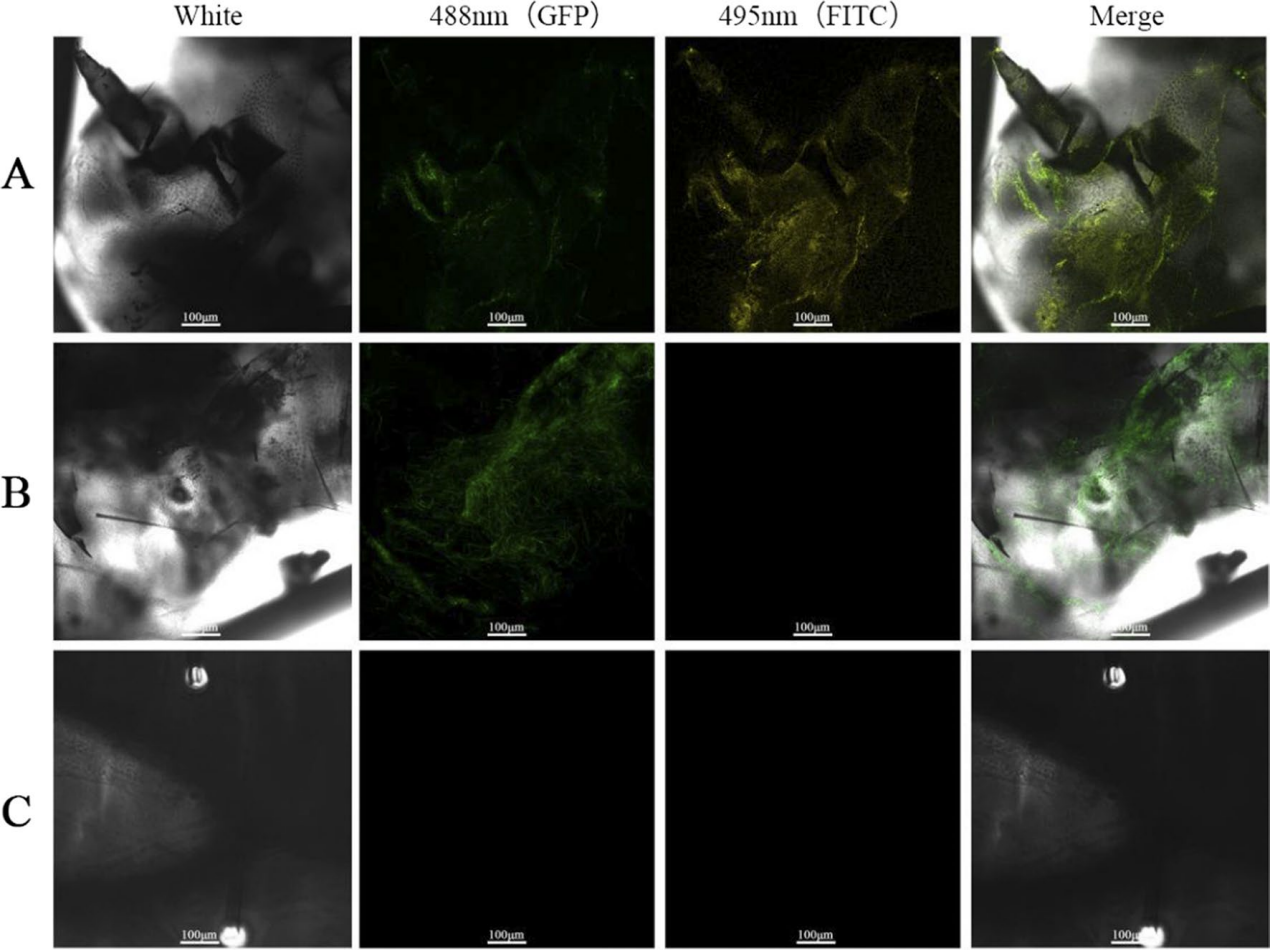
Fluorescence observation of virus distribution in insect bodies. **A** Insect bodies infected with virus-harbouring *B. bassiana* strains; **B** Insect bodies infected with virus-free *B. bassiana* strains; **C** Insect bodies without *B. bassiana* infection

The virus BbCV2 distribution in insect bodies following host fungi infection was also determined by immunofluorescence assay. The results showed that after infection with the virus-infected *B. bassiana* strain, at 488 nm and 495 nm, hyphae of *B. bassiana* and virions were clearly observed. Furthermore, for merged 488 nm and 495 nm wavelengths, either hyphae of *B. bassiana* orvirions could be observed, with virions distributed around mycelia of *B. bassiana*, showing that the virus was secreted from host fungi cells following infection (Fig. 4A). For samples infected with the virus-free *B. bassiana* strain, only hyphae of *B. bassiana* were visible and virions could not be observed (Fig. 4B). For samples without *B. bassiana* infection, neither hyphae of *B. bassiana* nor virions were visible (Fig. 4C).

### Virus BbCV2 particles infect *B. bassiana * in vitro

**Fig. 5:**
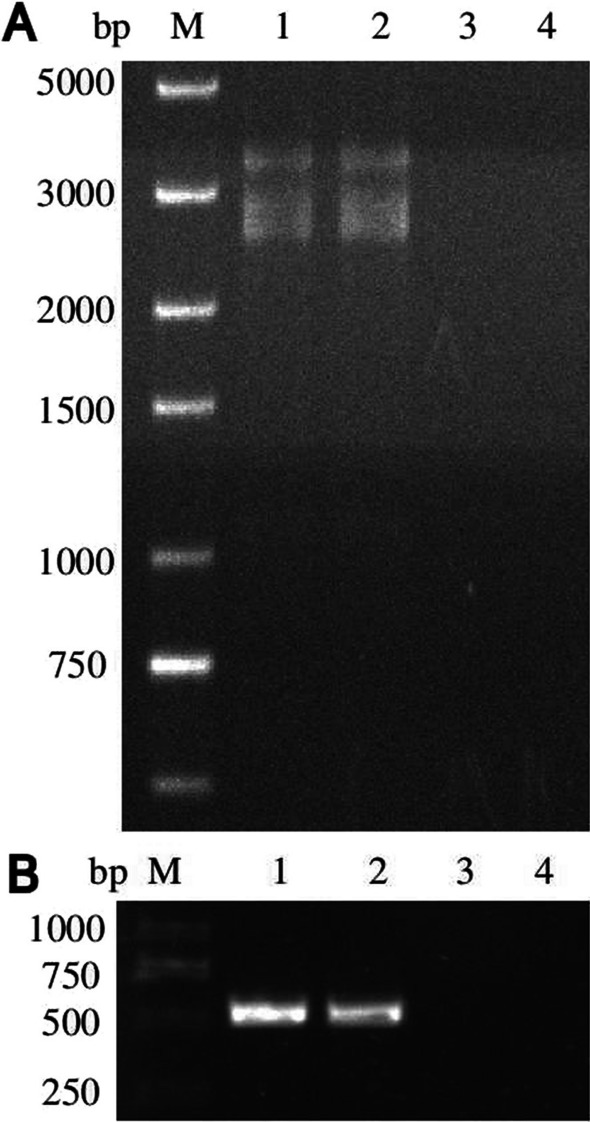
Detection of BbCV2 virionsinfection via co-incubation with *Beauveria bassiana* blastsporesby dsRNA extraction and RT-PCR. **A** dsRNA extraction. M, molecular marker; 1 and 2, *B. bassiana* mycelium co-incubation with virions; 3 and 4, control; **B** (**A**) RT-PCR. M, molecular marker; 1 and 2, *B. bassiana* mycelium co-incubation with virions; 3 and 4, control

Using the sucrose gradient method to extract BbCV2 virus particles, indirect ELISA results showed that the virus particles appeared within a 30% gradient of sucrose (Additional file 9: Fig. S4A) and were confirmed by the extraction of double stranded RNA (Additional file 9: Fig. S4B). After co-incubation of the virus particles with the *B. bassiana* blastspores, it was able to observe the presence of dsRNA in the mycelium (Fig. 5A), the RT-PCR identification showed that the dsRNA virus was BbPmV-4 (Fig. 5B). However, there was no dsRNA and RT-PCR target products in the mycelium of individually cultivated *B. bassiana*, which mean that the particles of virus BbCV2 had ability to infect *B. bassiana * in vitro.

### BbCV2 infection causes fungal host hypovirulence

**Fig. 6:**
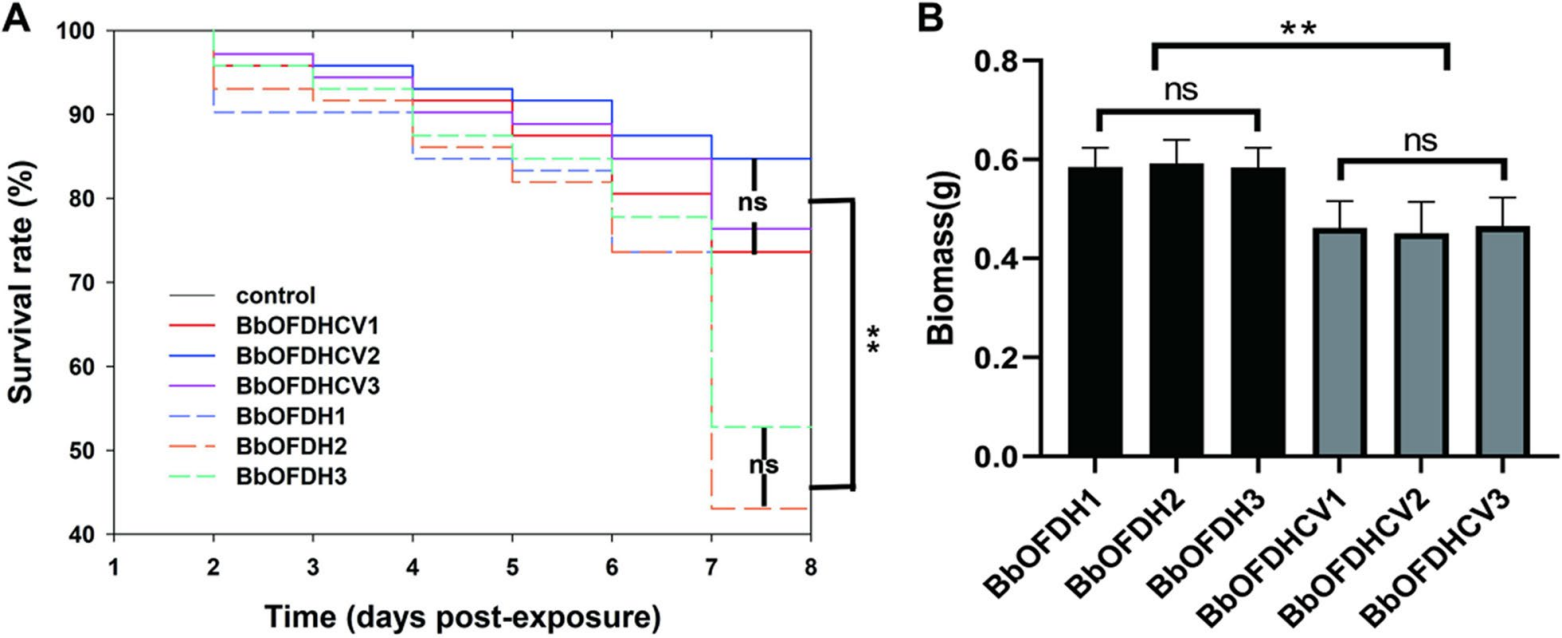
Virulence and biomass evaluation of *B. bassiana* strains with and without BbCV2 infection. **A** Evaluation of virus-infected and virus-free *B. bassiana* strains; **B** Biomass of virus-infected and virus-free *B. bassiana* strains. Error bars indicate standard deviation; ns = no significant difference; ** = significant difference. Duncan’s multiple range test (*p* < 0.01)

Infection with BbCV2 virus caused the hypovirulence and biomass decline of *B. bassiana*. The survival rate of *O. furnacalis* larvae infected with three virus-free strains and three virus-infected strains was significantly higher than that of virus-infected strains post-inoculation from 3 to 8 days. Thus, compared with virus-free strains BbOFDH1, BbOFDH2 and BbOFDH3, the virulence of virus-infected strains BbOFDHCV1, BbOFDHCV2 and BbOFDHCV3 was decreased significantly (Fig. 6A), and the biomass of the virus-free strains was significantly higher than that of the virus-infected strains, there were no differences within the group (Fig. 6B).

### Transcriptome analysis of virus-infected *B. bassiana*

#### Overview of RNA-Seq data

**Fig. 7:**
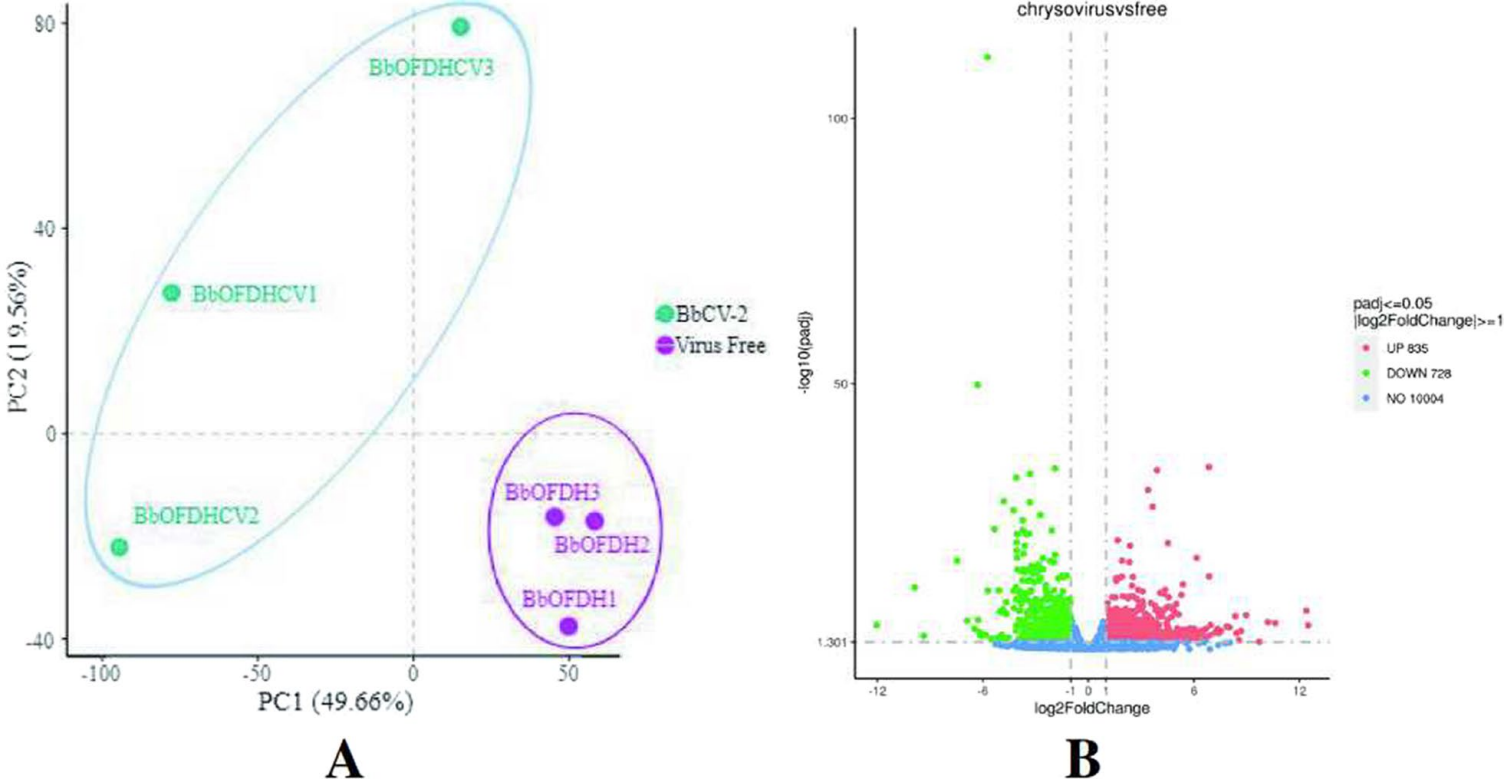
Transcriptome profile of RNA-Seq data. **A** Principal component analysis of virus-free and BbCV2 groups; **B** Volcano plot of RNA-Seq data using log_2_ fold change and log_10_ *p*-value. Red and green dots denote up- and downregulated genes, respectively, and blue dots indicate genes with no significant expression

After filtering and checking the raw data (raw reads) for sequencing error rates and GC content distribution, the yielded 40.32 Gb of clean data (clean reads) was used for subsequent analysis. It showed that all libraries had a Q20 value > 97% and Q30 > 93%. The percentage of total sequencing reads successfully matching the genome of Bb2860 exceeded 94%, confirming the quality and accuracy of the data (Additional file 10: Table S6). Correlation coefficients were calculated from the FPKM values of all genes in each sample and plotted as heatmaps. The intra-group R^2^ for virus-free strains BbOFDH1, BbOFDH2, and BbOFDH3, and virus-infected strainsBbOFDHCV1, BbOFDHCV2 and BbOFDHCV3 were all > 0.72 and close to 1 (Additional file 11: Fig. S5). The values for three virus-freestrains were all similar in terms of principal component 1 (PC1) and PC2, which of the threevirus-infected strains were also similar for PC1 but different for PC2. However, they were also well separated from the virus-free group, indicating good biological reproducibility within groups (Fig. 7A). Following BbCV2 infection, *B. bassiana* yielded 1563 statistically significant DEGs, of which 835 were up-regulated (red dots in Fig. 7B) and 728 were down-regulated (green dots in Fig. 7B).

#### GO enrichment analysis of DEGs

**Fig. 8:**
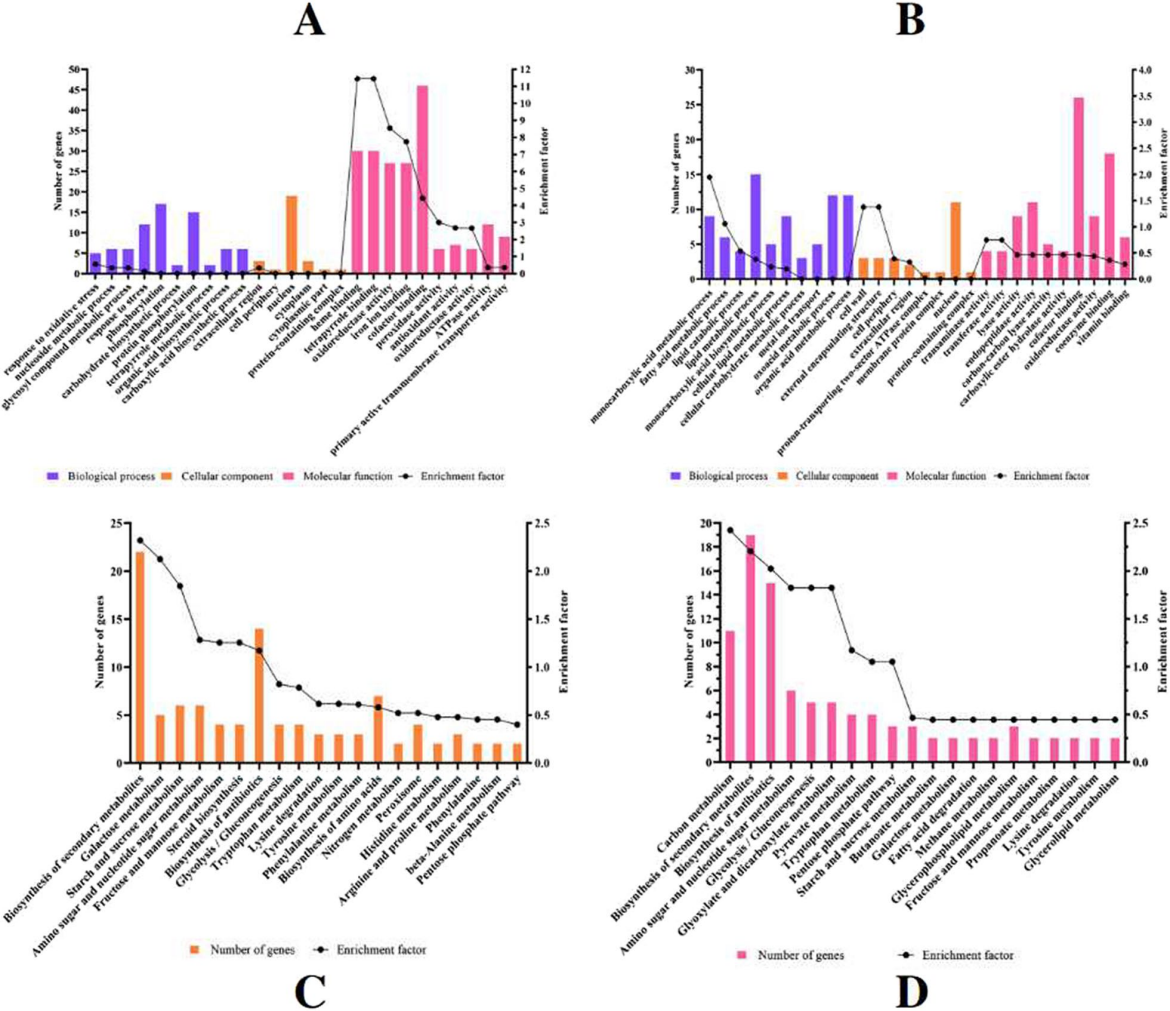
GO and KEGG enrichment analyses of DEGs. **A** GO enrichment analysis of upregulated genes; **B** GO enrichment analysis of downregulated genes; **C** KEGG enrichment analysis of upregulated genes; **D** KEGG enrichment analysis of downregulated genes

We performed GO analysis to define the underlying gene functions regulated by BbCV-2 infection. GO analysis of 835 upregulated genes showed that, in the biological process category, the GO terms “response to oxidative stress”, “nucleoside metabolic process”, “glycosyl compound metabolic process”, “phosphorylation”, “carbohydrate biosynthetic process”, “tetrapyrrole metabolic process”, “organic acid biosynthetic process” and “carboxylic acid biosynthetic process” were the most highly enriched (Fig. 8A). The main biological processes related to 728 down-regulated genes, were mainly focused on metabolic processes including “monocarboxylic acid metabolic process”, “lipid metabolic process”, “cellular lipid metabolic process”, “monocarboxylic acid biosynthetic process”, “cellular carbohydrate metabolic process” and so on (Fig. 8B).

#### KEGG enrichment analysis of DEGs

The DEGs were categorised using KEGG pathway analysis to assess the major pathways involved in fungus-mycovirus interactions. The 20 most significant KEGG pathways were selected for analysis. The upregulated genes were mainly enriched in “biosynthesis of secondary metabolites”, “galactose metabolism”, “starch and sucrose metabolism”, “amino sugar and nucleotide sugar metabolism” and “fructose and mannose metabolism” (Fig. 8C). The downregulated genes were mainly enriched in “carbon metabolism”, “biosynthesis of antibiotics”, “glyoxylate and dicarboxylate metabolism”, “pyruvate metabolism” and “pentose phosphate pathway” (Fig. 8D).

#### Effects of BbCV2 on virulence-related genes of B. bassiana

The epicuticle of insects is rich in proteins, chitin and lipids. The expression of some genes encoding functional enzymes that were responsible for the insect cuticle degradation in process of *B. bassiana* infection. For genes related to lipids degradation, the fasciclin domain-containing proteins-encoding genes *BBA_00614* and *BBA_05349* [42–44], and fatty acid hydroxylase superfamily protein-encoding genes *BBA_08427*, *BBA_06832*, *BBA_05271*, *BBA_03495*, *BBA_03308* and *BBA_02581* [45] were significantly downregulated following BbCV2 infection of *B. bassiana*. It has been proved that both enzymes subtilisin-like protease (Pr1) and chitinases and cytochrome P450 (CYP) metabolizeby *B. bassiana* contributed to penetration of the insect cuticle [45–47]. Expression of the *Pr1* and chitinase-related gene *BBA_04617*, as well as genes encoding CYP52 and other CYPs, including *BBA_03806*, *BBA_06473*, *BBA_08111* and *BBA_09022*, were also significantly downregulated following BbCV2 infection. Mitogen-activated protein kinase (MAPK) participates in the process by enhancing the ability to penetrate the insect cuticle from inside to outside [48], here, the expression of MAPK-related gene *BBA_09043* was significantly downregulated in the BbCV2 infected strain, although its function needs to be clarified.

After penetrating the insect epidermis, *B. bassiana* forms hyphae within the insect body, which not only cause mechanical damage, but also metabolize toxins to kill the insect. It has been proved that AMAP kinase BBSLT2 encoding gene *BBA_03334* could control growth, conidiation, cell wall integrity, and virulence of *B. bassiana* [49], which was significantly down-regulated following the BbCV2 infection. Jin et al. (2021) found that the knockout of two biomass related genes *Bbpyr* (*Bb_03937*) and *Bbthi* (*Bb_04964*) could result in the poor colony growth, fewer hyphae and conidia of *B. bassiana* [50], herein, the expression of both the two genes in virus-infected strains were significantly lower than that of the virus-free strains. Previous studies have shown that the BCS1-domain containing proteins were significantly involved in fungal growth and virulence [51], in this study, three genes *BA_03426*, *BBA_10003* and *BBA_09074* encoding homologs of BCS1 domain-containing proteins were significantly down-regulated following the virus infection.

Polyketide synthases (PKSs) synthesize secondary metabolites such as oosporein that inhibit polyphenol oxidase (PPO) activity, which leads to inhibition of the expression of insect antimicrobial peptides [52, 53]. In the present study, PKS-related genes *BBA_06613*, *BBA_03616* and *BBA_09856* were significantly downregulated. Nonribosomal peptide synthases (NRPSs) are closely related to the metabolism of microbial toxins and antibiotics. *B. bassiana* can metabolise various toxins through NRPS pathways, including beauveritin 14, 2-pyridone tenellin, bassianin 25, beauveritin and bassianolide [54–56]. Three virulence-related NRPS genes (*BBA_04028*, *BBA_08222* and *BBA_03671*) were identified in *B. bassiana* isolated from *Plutella xylostella* [57]. Here, we identified four NRPS-related genes (*BBA_09856*, *BBA_03616*, *BBA_01841* and *BBA_04028*) that were significantly downregulated. *BBA_04028* encodes BbLaeA, and knockout of *BBA_04028* diminished beauvericin and bassiatin metabolism, while overexpression of *BbLaeA* increased the production of toxins [58].

#### Gene expression level determination by qRT-PCR

To validate the results obtained in RNA-Seq experiments, four upregulated and six downregulated genes were randomly selected for qRT-PCR validation with specific primers. Relative expression measured by qRT-PCR was consistent with expression levels measured by RNA-seq for all tested genes (Additional file 12: Fig. S6), confirming that the transcriptome data were reliable.

## Discussion

Mycoviruses can not only reduce the pathogenicity of phytopathogenic fungi but also convert host pathogenic fungi into a biocontrol agent [54, 59]. Moriyama et al. have reported that Magnaporthe oryzae chrysovirus 1 strains A and B (MoCV1-A and MoCV1-B) were the mycoviruses that cause hypovirulence traits in their host fungus *Magnaporthe oryzae* [60]. It has been found that multiple mycoviruses caused hypovirulence of Botrytis cinerea strains, including Botrytis cinerea RNA virus 1 (BcRV1), B. cinerea CCg378 virus 1 (Bc378V1), Botrytis cinerea hypovirus 1 (BcHV1) and Botrytis cinerea fusarivirus 1 (BcFV1) etc. [61–63]. Guo et al. reported two mycoviruses Colletotrichum fructicola ourmia-like virus 1-Colletotrichum gloeosporioides ourmia-like virus 1 (CfOLV1-CgOLV1) and Colletotrichum fructicola ourmia-like virus 2 (CfOLV2), which infection caused hypovirulence of *Colletotrichum fructicola*, a phytopathogenic fungus causing leaf black spot and fruit rot disease in a wide variety of crops [64]. Zhong et al. found that a Hypovirulence-associated mycoviruses *Sclerotinia sclerotiorum* hypovirus 2 (SsHV2) had great potential and application prospects for controlling the fungal diseasesouthern blight in a wide variety of crops, which caused by a phytopathogens *Sclerotium rolfsii* [65]. Borwmann et al. have not only found that the Fusarium graminearum virus China 9 (FgV-ch9) was a hypovirulence-associated mycovirus to *Fusarium graminearum*, the causal agent of fusarium head blight, but also further clarified that virus response 1 (vr1) was a key hypovirulence causing factor of the virus (expression of a structural protein of the mycovirus FgV-ch9 negatively affects the transcript level of a novel symptom alleviation factor and causes virus infection-Like Symptoms in *F. graminearum*) [66]. Unfortunately, mycoviruses can also hypovirulence of entomopathogenic fungi. Wang et al. (2023) found that infection with partitivirus Metarhizium majus partitivirus 1 (MmPV1) decreased conidiation and tolerance to heat shock and UV-B irradiation, and especially hypovirulence toward the insect pest of the host entomogenous fungus *Metarhizium majus* [67]. However, there have been few reports on mycoviruses causing *B. bassiana* hypovirulence. In the present study, we found that mycovirus BbCV2 caused hypovirulence of *B. bassiana* toward the insect pest *O. furnacatis*, and decreased biomass.

Decreased pathogenicity of *B. bassiana* during field usage and strain preservation affect its biocontrol applications. Epidemics of mycoviruses in phytopathogenic fungi provide a biological control opportunity for plant diseases. However, whether mycoviruses that infect entomopathogenic fungi are prevalent in the field and can thereby cause hypovirulence of wild *B. bassiana* remains unknown. Here, we collected and characterised *B. bassiana* strains from diseased *O. furnacatis* in different geographical locations in fields in Jilin Province, China, and discovered that BbCV2 virus infection was widely present in wild *B. bassiana* strains, with a virus-harbouring rate up to 90%. Furthermore, the virus-harbouring proportion was not correlated with collection site, indicating that the virus is likely to spread naturally within the region. Jia et al. monitored the interannual dynamics and abundance of mycovirus infections in *S. sclerotiorum*; 24 kinds of mycoviruses classified as members of the local core virus group exhibited persistence and relatively high transmissibility in a single crop field [68]. In the present study, the BbCV2 virus was the core virus infecting *B. bassiana* isolated from *O. furnacatis* from different sites, and epidemics of this virus may be a key factor responsible for hypovirulence of *B. bassiana* in the field.

During interactions with host fungi, mycoviruses can mutate, leading to genetic diversity. Viral genetic diversity has important implications for virus persistence, pathogenesis and transmission [69], and is the result of long-term interactions between heritability including mutation, gene recombination, gene flow (gene migration), random drift and natural selection [70]. Previous studies demonstrated that the RdRp gene and its protein product are the only universal gene and protein among RNA viruses, making them primary targets for genetic diversity analysis of RNA viruses [71, 72]. In the present study, there was no significant difference in the RdRp base content of the tested BbCV2 viruses from different collection sites, and the G + C content was higher than the A + T content. A higher G + C base content can endow the nucleic acid structure with greater stability and reduce genetic variations such as mismatches. No minimalist information sites were found, and phylogenetic tree analysis revealed high amino acid sequence identity, further indicating that viruses were genetically stable in their natural environments, and that the host fungus *B. bassiana* places no significant selection pressure on the virus, suggesting that the virus takes the initiative in interactions between the two.

Mycoviruses can transmit interspecifically and intraspecifically between host fungi [73, 74]. Liu et al. found that SsHADV-1 could not only infect its host fungus extracellularly, but also the mycophagous insect *Lycoriella ingenua*, which was regarded as a transmission vector for virus spread, and more importantly, this virus could multiply and transmit vertically within the life cycle of insects [75]. Urayama et al. found that mycoviruses S-0412-II 1a affected vegetative growth in the rice blast fungus *Magnaporthe oryzae*, which was detected not only in host cells but also in culture supernatant. Furthermore, abnormal aggregation of mycelia was observed after adding the mycovirus-containing culture supernatant to an uninfected strain of *M. oryzae* and mycoviral dsRNAs were detectable from the aggregated mycelia [76]. Our previous research found that BbCV2 and BbPmV-4 could spread from *B. bassiana* to another two *Beauveria* species via not only hyphal anastomosis but also insect coinfection [28]. In the present study, we observed efficient transmission from virus-infected strains to virus-free strains of *B. bassiana*, as well as vertical transmission in the same strain. However, it is not clear whether the virus transmitted through direct fusion of mycelia by coculture on medium and coinfection in insect bodies, or whether virions could be released outside host fungi cells and then infect other strains. Here, we detected the viruses BbCV2 and BbPmV-4 both inside host fungal cells and in the culture medium by indirect-ELISA, the viruses concentration in the supernatant are higher than that of fungal cells, which confirmed that the mycoviruses had the ability to dissociate outside the cells of host fungi, and the virions can also infect *B. bassiana* blastspores through co-incubation, it was consistent with the research of Urayama et al., in which they found that purified virions could infect host fungi in vitro [76]. Furthermore, the virus BbCV2 could be secreted into the insect body after infection of the host fungal strain, as determined by both indirect-ELISA and immunofluorescence assay, indicating that BbCV2 may be escape from the original host and subsequently infect new fungal hosts and spread, leading to virus epidemics within the *B. bassiana* population, thereby diminishing the virulence of wild *B. bassiana* strains in the field. Further research is needed to explore whether the BbCV2 mycovirus can infect insects and multiply in the insect body.

In studies on the interaction mechanisms between mycoviruses and host fungi, transcriptome data are important for clarifying the influence of viruses on the main functional genes and pathways of hosts, and transcriptomics is widely used to screen interacting genes. In *B. cinerea* infected by Botryosphaeria dothidea chrysovirus 1 (BdCV1) and Botryosphaeria dothidea partitivirus 1 (BdPV1) viruses, differential genes between virus-infected and virus-free strains were identified, and DEGs enrichment analysis identified genes related to metabolic processes, cellular processes, catalytic activity, transporter activity, signal transduction and other biological pathways, and subsequent KEGG analysis identified numerous DEGs associated with metabolism, transcription and signal transduction [77]. In *S. sclerotiorum*, 958 mRNAs were affected by the hypovirulent hypovirus2-L, with over 100 genes involved in basic metabolism and sugar and lipid transport [78]. Pathogenicity of *B. bassiana* is a complex biological process involving the metabolism of hydrolytic enzymes for epidermis penetration [42], the hypha growth, andthe toxins metabolizedby hyphae after the insect epidermispenetration [43]. In our previous research, transcriptome analysis of *B. bassiana* infected with hypervirulent mycovirus BbPmV-4 showed that virus infection significantly increased the expression levels of some genes related to MAPK (*BBA_09022*) and cytochrome P450 (*BBA_03806*, *BBA_06473* and *BBA_08111*) in *B. bassiana* [79]. By contrast, these genes were downregulated after BbCV2 infection. Furthermore, some genes related PKS and NRPS pathways were identified in this study. In addition, biomass was significantly decreased following virus infection, which likely causes a decrease of physical damage and toxin metabolism. The hypovirulence mechanism of partitivirus MmPV1 toward host fungi was known to involve a decrease in toxin metabolism caused by infection, including effects on triterpenoids and metarhizins A and B [67]. Hence, interactions between mycoviruses and genes/pathways related to insect epidermis penetration, fungal biomass and toxin metabolism may be involved in the mechanisms by which BbCV2 causes hypovirulence of *B. bassiana*, but this needs to be verified at the protein level though co-immunoprecipitation assays [80, 81] and pull-down assays [82], both of which are efficient methods for probing virus-host interactions. Here, a polyclonal antibody with high titre and specificity against mycovirus BbCV2 coat protein was prepared, providing a useful tool for investigating interactions between BbCV2 and its *B. bassiana* fungal host in future.

In the past, acquisition of high-virulence strains of *B. bassiana* has mainly involved isolation and screening of wild strains collected from the field using biological testing, which is time-consuming and labour-intensive [83, 84]. Here, we found that mycoviruses may be a key factor causing the decline of *B. bassiana* virulence, and virus detection could be used to exclude hypovirulent virus-infected strains, and to screen strains of wild *B. bassiana* with high virulence, thereby improving the efficiency of high virulence strain screening. To the best of our knowledge, detection of mycoviruses has mainly been performed using dsRNA and RT-PCR methods, which require complex software and can be used for qualitative but not quantitative testing. Efficient and specific serological techniques have been widely used for quantitative and qualitative detection of animal and plant viruses [85, 86], but there are few reports that they have been applied for detection of mycoviruses. Here, we prepared two highly efficient and specific polyclonal antibodies against mycoviruses BbCV2 and BbPmV-4 coat proteins, and established a serological system that expands the detection methods available for mycoviruses, especially for quantitative detection.

### Supplementary Information

**Additional file 1: Table S1.** Tested strains

**Additional file 2: Table S2.** Primers for RdRp gene amplification

**Additional file 3: Table S3.** Base content of RdRp gene sequences of BbCV2 viruses

**Additional file 4: Table S4.** Sequence information

**Additional file 5: Table S5.** Efficiency of virus vertical transmission of *B. bassiana* via subculture

**Additional file 6: Fig. S1.** SDS-PAGE analysis of purified recombinant BbPmV-4-CP and BbCV2-CP proteins. (A) Ultrafiltration of recombinant BbPmV-4-CP Protein. Lane 1, recombinant BbPmV-4-CP protein following ultrafiltration. (B) Ultrafiltration of recombinant BbCV2-CP Protein. Lane 1-5, recombinant BbCV2-CP protein following ultrafiltration.

**Additional file 7: Fig. S2.** Determination of the polyclonal antibody titre of BbPmV-4-CP and BbCV2-CP protein. (A) BbPmV-4-CP. (B) BbCV2-CP.

**Additional file 8: Fig. S3.** Detection of BbCV2 and BbPmV-4 virus in and outside host cells by indirect-ELISA. (A) Liquid culture medium of *Beauveria bassiana* containing virus BbCV2.1, blank control; 2, positive control (BbCV2-CP); 3, negative control (supernatant of virus-free strains); 4, supernatant of BbCV2 virus-harbouring strains; 5, negative control (pellet of virus-free strains); 6, pellet of BbCV2 virus-harbouring strains; (B) Liquid culture medium of *Beauveria bassiana* containing virus BbPmV-4.1, blank control; 2, positive control (BbPmV-4-CP); 3, negative control (supernatant of virus-free strains); 4, supernatant of BbPmV-4 virus-harbouring strains; 5, negative control (pellet of virus-free strains); 6, pellet of BbPmV-4 virus-harbouring strains. (C) Insect bodies. 1-2, negative control (larvae without *B. bassiana* infection); 3-4, larvae infected by BbOFDH; 5-6, larvae infected by BbOFDHCV; 7, positive control (BbCV2-CP).

**Additional file 9: Fig. S4.** Detection of BbCV2 virions by indirect-ELISA and dsRNA extraction. (A) Detection of virions in different gradients of sucrose by indirect-ELISA. +, positive control (BbCV2-CP); −, blank control; 20%–50%, different gradients of sucrose; (B) DsRNA of virions in 30% sucrose.

**Additional file 10: Table S6.** Summary of sequencing data

**Additional file 11: Fig. S5.** Correlation heatmap of strain samples

**Additional file 12: Fig. S6.** qRT-PCR verification of RNA-Seq gene expression levels

## Data Availability

All raw data of RNA-seq are available at Sequence Read Archive (PRJNA822034 and PRJNA993422). All materials can be obtained from corresponding author.
