# Supplementary material for: Hypovirulence-associated mycovirus epidemics cause pathogenicity degeneration of Beauveria bassiana in the field
Source: Virol J. 2023 Nov 3;20:255. doi: 10.1186/s12985-023-02217-6 (PMC10623766; doi:10.1186/s12985-023-02217-6)
Supplement: Supplementary file 1 — Additional file 1: Table S1. Tested strains [file 12985_2023_2217_MOESM1_ESM.docx]

**Table S6: Summary of sequencing data**

| **Sample** | **Raw reads** | **Clean reads** | **Clean bases** | **Q20 (%)** | **Q30 (%)** | **Reads mapped to *B. bassiana* genome** | **Mapping rate** |
| --- | --- | --- | --- | --- | --- | --- | --- |
| BbOFDH1 | 47180942 | 46864626 | 7.03G | 97.61 | 93.66 | 45026982 | 96.08% |
| BbOFDH2 | 44683726 | 43994434 | 6.60G | 98.19 | 94.84 | 41859899 | 95.15% |
| BbOFDH3 | 41238812 | 40573572 | 6.09G | 98.16 | 94.78 | 38331907 | 94.48% |
| BbOFDHCV1 | 46265966 | 44754776 | 6.71G | 98.29 | 95.05 | 42809722 | 95.65% |
| BbOFDHCV2 | 46116880 | 44110758 | 6.62G | 98.24 | 94.93 | 42054314 | 95.34% |
| BbOFDHCV3 | 49690024 | 48457262 | 7.27G | 98.05 | 94.52 | 46321989 | 95.59% |
