# Supplementary figures and images for: Hypovirulence-associated mycovirus epidemics cause pathogenicity degeneration of Beauveria bassiana in the field
Source: Virol J. 2023 Nov 3;20:255. doi: 10.1186/s12985-023-02217-6 (PMC10623766; doi:10.1186/s12985-023-02217-6)

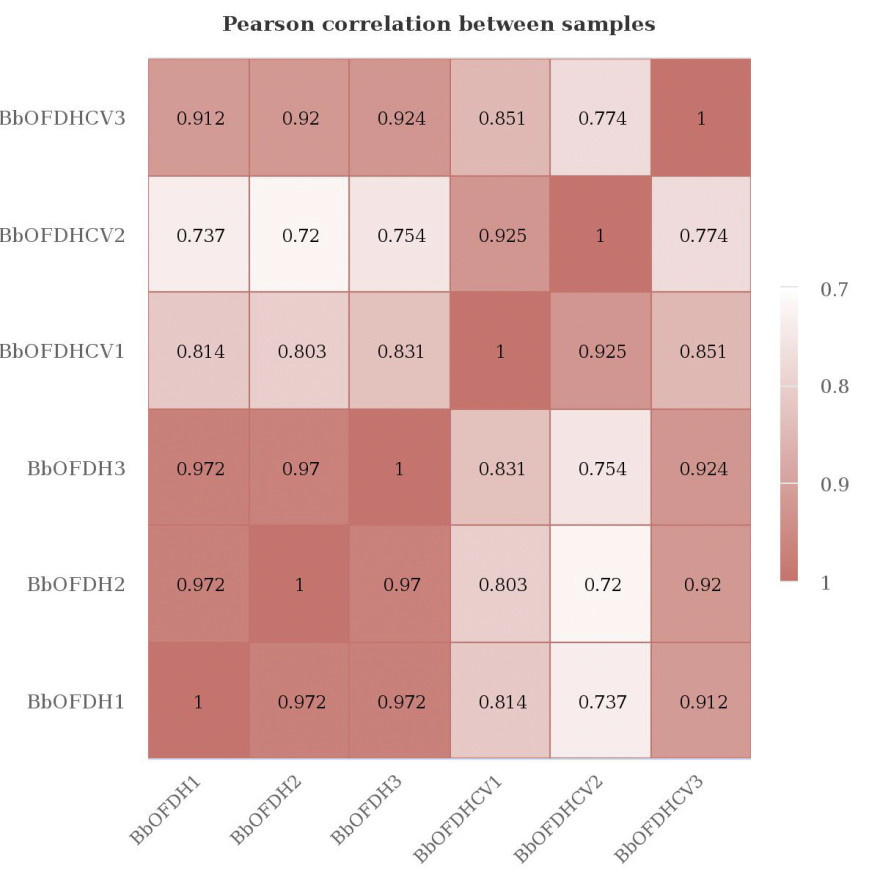


**Figure S5 Correlation heatmap of strain samples**

Supplement: Supplementary file 2 — Additional file 2: Table S2. Primers for RdRp gene amplification [file 12985_2023_2217_MOESM2_ESM.docx]

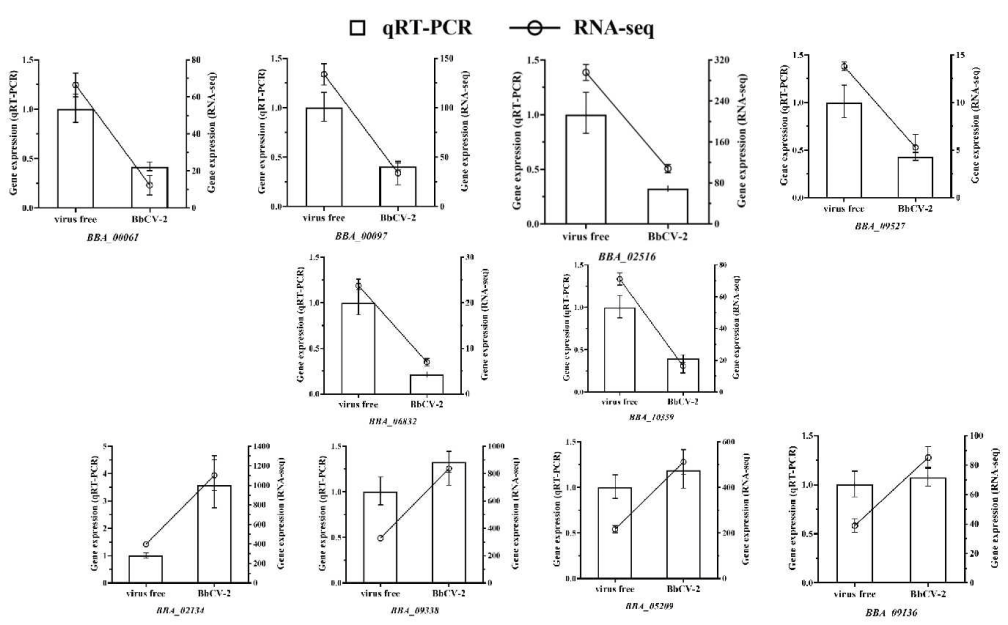


**Fig. S6 qRT-PCR verification of RNA-Seq gene expression levels**

Supplement: Supplementary file 3 — Additional file 3: Table S3. Base content of RdRp gene sequences of BbCV2 viruses [file 12985_2023_2217_MOESM3_ESM.docx]
