# Supplementary material for: Hypovirulence-associated mycovirus epidemics cause pathogenicity degeneration of Beauveria bassiana in the field
Source: Virol J. 2023 Nov 3;20:255. doi: 10.1186/s12985-023-02217-6 (PMC10623766; doi:10.1186/s12985-023-02217-6)
Supplement: Supplementary file 4 — Additional file 4: Table S4. Sequence information [file 12985_2023_2217_MOESM4_ESM.docx]

**Table S1 Tested strains**

| Collection locations | Geographical coordinates | Codes | Numbers of stains |
| --- | --- | --- | --- |
| Baishan | E 128°11'21", N41°24'32" | BS | 21 |
| Fusong | E 127°48'41", N42°17'55" | FS | 12 |
| Yongji | E 126°50'44", N43°67'87" | YJ | 15 |
| Antu | E 129°48'11", N42°21'18" | AT | 15 |
| Changling | E 123°96'75", N44°27'59" | CL | 16 |
| Lishu City | E 123°46'28", N43°22'30" | LS | 13 |
| Dongfeng | E 125°53'10", N42°67'69" | DF | 14 |
