# Supplementary material for: Hypovirulence-associated mycovirus epidemics cause pathogenicity degeneration of Beauveria bassiana in the field
Source: Virol J. 2023 Nov 3;20:255. doi: 10.1186/s12985-023-02217-6 (PMC10623766; doi:10.1186/s12985-023-02217-6)
Supplement: Supplementary file 5 — Additional file 5: Table S5. Efficiency of virus vertical transmission of B. bassiana via subculture [file 12985_2023_2217_MOESM5_ESM.docx]

**Table S2 Primers for RdRp gene amplification**

| Primer name | Sequence (5´−3´) |
| --- | --- |
| RCV1-2F | CTACCGCAAAAAAGAGAAAAAAGC |
| RCV1-2R | GGTCAGCGATCTCCTTAGCC |
| RCV2-2F | GACGTGGGCCCGGATAATAC |
| RCV2-2R | AGCCGGGTCTACAAACTTCG |
| RCV3-2F | ATTCGGGCATGGGATGTTGT |
| RCV3-2R | CGGTAGATTGTTGTTGCAATCTG |
