# Supplementary material for: Hypovirulence-associated mycovirus epidemics cause pathogenicity degeneration of Beauveria bassiana in the field
Source: Virol J. 2023 Nov 3;20:255. doi: 10.1186/s12985-023-02217-6 (PMC10623766; doi:10.1186/s12985-023-02217-6)
Supplement: Supplementary file 6 — Additional file 6: Fig. S1. SDS-PAGE analysis of purified recombinant BbPmV-4-CP and BbCV2-CP proteins. (A) Ultrafiltration of recombinant BbPmV-4-CP Protein. Lane 1, recombinant BbPmV-4-CP protein following ultrafiltration. (B) Ultrafiltration of recombinant BbCV2-CP Protein. Lane 1-5, recombinant BbCV2-CP protein following ultrafiltration. [file 12985_2023_2217_MOESM6_ESM.docx]

**Table S3 Base content of RdRp gene sequences of BbCV2 viruses**

| **Regions** | **A** | **U** | **C** | **G** | **A+U** |
| --- | --- | --- | --- | --- | --- |
| AT | 21.61% | 20.09% | 25.38% | 32.91% | 41.70% |
| BS | 21.61% | 20.11% | 25.36% | 32.91% | 41.72% |
| CL | 21.61% | 20.10% | 25.38% | 32.90% | 41.71% |
| DF | 21.61% | 20.10% | 25.38% | 32.90% | 41.71% |
| FS | 21.61% | 20.11% | 25.36% | 32.91% | 41.72% |
| LS | 21.57% | 19.98% | 25.38% | 33.06% | 41.55% |
| YJ | 21.61% | 20.09% | 25.38% | 32.91% | 41.70% |
| Average | 21.60% | 20.08% | 25.37% | 32.93% | 41.69% |
