# Supplementary material for: Hypovirulence-associated mycovirus epidemics cause pathogenicity degeneration of Beauveria bassiana in the field
Source: Virol J. 2023 Nov 3;20:255. doi: 10.1186/s12985-023-02217-6 (PMC10623766; doi:10.1186/s12985-023-02217-6)
Supplement: Supplementary file 7 — Additional file 7: Fig. S2. Determination of the polyclonal antibody titre of BbPmV-4-CP and BbCV2-CP protein. (A) BbPmV-4-CP. (B) BbCV2-CP. [file 12985_2023_2217_MOESM7_ESM.docx]

**Table S4 Sequence information**

| **Number** | **Gene name** | **Database** | | **Access number** |
| --- | --- | --- | --- | --- |
| 1 | Cherry chlorotic rusty spot associated chrysovirus RdRp | | GenBank | AJ781397 |
| 2 | Aspergillus fumigatus chrysovirus segment 1 | | GenBank | NC_038872.1 |
| 3 | Cryphonectria nitschkei chrysovirus 1 RdRp gene | | GenBank | LC062729.1 |
| 4 | Fusarium oxysporum chrysovirus 1 putative RNA polymerase gene | | GenBank | NC_043218.1 |
| 5 | Helminthosporium victoriae 145S virus segment 1 RNA-dependent RNA polymerase mRNA | | GenBank | AF297176.1 |
| 6 | Penicillium chrysogenum virus segment 1 RNA-dependent RNA polymerase gene | | GenBank | AF296439.1 |
| 7 | Verticillium dahliae chrysovirus 1 segment 1 | | GenBank | NC_038784.1 |
| 8 | Anthurium mosaic-associated virus strain PHA segment dsRNA1 | | GenBank | NC_043676.1 |
| 9 | Brassica campestris chrysovirus 1 isolate Hubei segment RNA1 | | GenBank | NC_043660.1 |
| 10 | Colletotrichum gloeosporioides chrysovirus 1 isolate HZ-1 segment 1 RNA-directed RNA-polymerase (RdRp) gene | | GenBank | NC_043666.1 |
| 11 | Raphanus sativas chrysovirus 1 segment RNA1 putative RNA-dependent RNA polymerase gene | | GenBank | JQ045335.1 |
| 12 | Persea americana chrysovirus segment RNA1 | | GenBank | NC_043506.1 |
| 13 | Botryosphaeria dothidea chrysovirus 1 isolate LW-1 segment RNA 1 putative RNA-dependent RNA polymerase (RdRp) gene | | GenBank | KF688736.1 |
| 14 | Fusarium graminearum dsRNA mycovirus-2 isolate 98-8-60 segment RNA 1 | | GenBank | NC_055221.1 |
| 15 | Fusarium graminearum mycovirus-China 9 segment 1 | | GenBank | HQ228213.1 |
| 16 | Fusarium oxysporum f. sp. dianthi mycovirus 1 isolate Fod116 segment dsRNA1 | | GenBank | KP876629.1 |
| 17 | Magnaporthe oryzae chrysovirus 1-A RdRp gene for RNA dependent RNA polymerase | | GenBank | NC_014462.1 |
| 18 | Magnaporthe oryzae chrysovirus 1 B RdRp gene for RNA dependent RNA polymerase | | GenBank | NC_023041.1 |
| 19 | Aspergillus mycovirus 1816 putative RNA-dependent RNA polymerase gene | | GenBank | EU289896.1 |
| 20 | Tolypocladium cylindrosporum virus 2 RdRp gene for RNA dependent RNA polymerase | | GenBank | FR750563.1 |
| 21 | Isaria javanica chrysovirus 1 isolate IjCV-1 RNA-dependent RNA polymerase (RdRp) gene | | GenBank | NC_033277.1 |
| 22 | Macrophomina phaseolina chrysovirus 1 isolate TN263 RNA-dependent RNA polymerase gene | | GenBank | NC_043662.1 |
| 23 | Agaricus bisporus virus 1 (ABV1) L1 dsRNA | | GenBank | X94361.1 |
| 24 | Alternaria alternata chrysovirus 1 AaCV1 RNA | | GenBank | NC_040738.1 |
| 25 | Bipolaris maydis chrysovirus 1 strain SS01 segment RNA1 | | GenBank | KY489954.1 |
| 26 | Grapevine associated chrysovirus-1 isolate Ctg446 putative RNA-dependent RNA polymerase gene | | GenBank | GU108588.1 |
| 27 | Penicillium janczewskii chrysovirus 1 RNA 1 | | GenBank | NC_028495.1 |
| 28 | Penicillium janczewskii chrysovirus 2 segment RNA1 ORF1 gene | | GenBank | NC_043672.1 |
| 29 | Shuangao chryso-like virus 1 RdRp gene | | GenBank | MH745159.1 |
| 30 | Wuhan insect virus 29 strain WHZM9836 hypothetical protein gene | | GenBank | KX882987.1 |
| 31 | Saccharomyces cerevisiae virus L-A (L1) putative RNA-dependent RNA polymerase gene | | GenBank | NC_003745.1 |
| 32 | Colletotrichum fructicola chrysovirus 1 | | GenBank | MG425969.1 |
