# Supplementary material for: Hypovirulence-associated mycovirus epidemics cause pathogenicity degeneration of Beauveria bassiana in the field
Source: Virol J. 2023 Nov 3;20:255. doi: 10.1186/s12985-023-02217-6 (PMC10623766; doi:10.1186/s12985-023-02217-6)
Supplement: Supplementary file 8 — Additional file 8: Fig. S3. Detection of BbCV2 and BbPmV-4 virus in and outside host cells by indirect-ELISA. (A) Liquid culture medium of Beauveria bassiana containing virus BbCV2.1, blank control; 2, positive control (BbCV2-CP); 3, negative control (supernatant of virus-free strains); 4, supernatant of BbCV2 virus-harbouring strains; 5, negative control (pellet of virus-free strains); 6, pellet of BbCV2 virus-harbouring strains; (B) Liquid culture medium of Beauveria bassiana containing virus BbPmV-4.1, blank control; 2, positive control (BbPmV-4-CP); 3, negative control (supernatant of virus-free strains); 4, supernatant of BbPmV-4 virus-harbouring strains; 5, negative control (pellet of virus-free strains); 6, pellet of BbPmV-4 virus-harbouring strains. (C) Insect bodies. 1-2, negative control (larvae without B. bassiana infection); 3-4, larvae infected by BbOFDH; 5-6, larvae infected by BbOFDHCV; 7, positive control (BbCV2-CP). [file 12985_2023_2217_MOESM8_ESM.docx]

**Table S5 Efficiency of virus vertical transmission of *B. bassiana* via subculture**

| **Strains** | **Virus-harbouring rate of the 2nd generation** | **Virus-harbouring rate of the 3rd generation** | **Significance of the differences** |
| --- | --- | --- | --- |
| BbOFDHCV1 | 73.33 ± 5.77% | 76.67 ± 5.77% | ns |
| BbOFDHCV2 | 80.00 ± 10.00% | 80.00 ± 0.00% | ns |
| BbOFDHCV3 | 76.67 ± 11.55% | 76.67 ± 5.77% | ns |
| Significance of differences | ns | ns |  |

Data were analysed using SPSS software ver. 26.0 (one-way ANOVA) and Duncan’s multiple range test (*p*<0.05). ns, no significant difference.
