# Supplementary material for: Hypovirulence-associated mycovirus epidemics cause pathogenicity degeneration of Beauveria bassiana in the field
Source: Virol J. 2023 Nov 3;20:255. doi: 10.1186/s12985-023-02217-6 (PMC10623766; doi:10.1186/s12985-023-02217-6)
Supplement: Supplementary file 9 — Additional file 9: Fig. S4. Detection of BbCV2 virions by indirect-ELISA and dsRNA extraction. (A) Detection of virions in different gradients of sucrose by indirect-ELISA. +, positive control (BbCV2-CP); −, blank control; 20%–50%, different gradients of sucrose; (B) DsRNA of virions in 30% sucrose. [file 12985_2023_2217_MOESM9_ESM.docx]

**
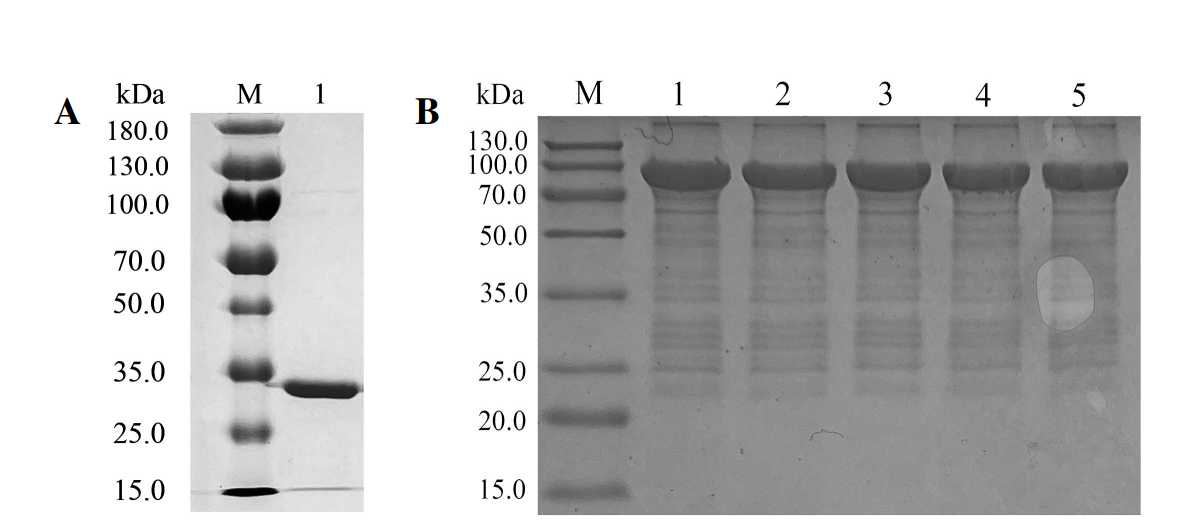
**

**Fig. S1 SDS-PAGE analysis of purified recombinant BbPmV-4-CP and BbCV2-CP proteins.** (A) Ultrafiltration of recombinant BbPmV-4-CP Protein. Lane 1, recombinant BbPmV-4-CP protein following ultrafiltration. (B) Ultrafiltration of recombinant BbCV2-CP Protein. Lane 1-5, recombinant BbCV2-CP protein following ultrafiltration.
