# Supplementary material for: Hypovirulence-associated mycovirus epidemics cause pathogenicity degeneration of Beauveria bassiana in the field
Source: Virol J. 2023 Nov 3;20:255. doi: 10.1186/s12985-023-02217-6 (PMC10623766; doi:10.1186/s12985-023-02217-6)
Supplement: Supplementary file 10 — Additional file 10: Table S6. Summary of sequencing data [file 12985_2023_2217_MOESM10_ESM.docx]

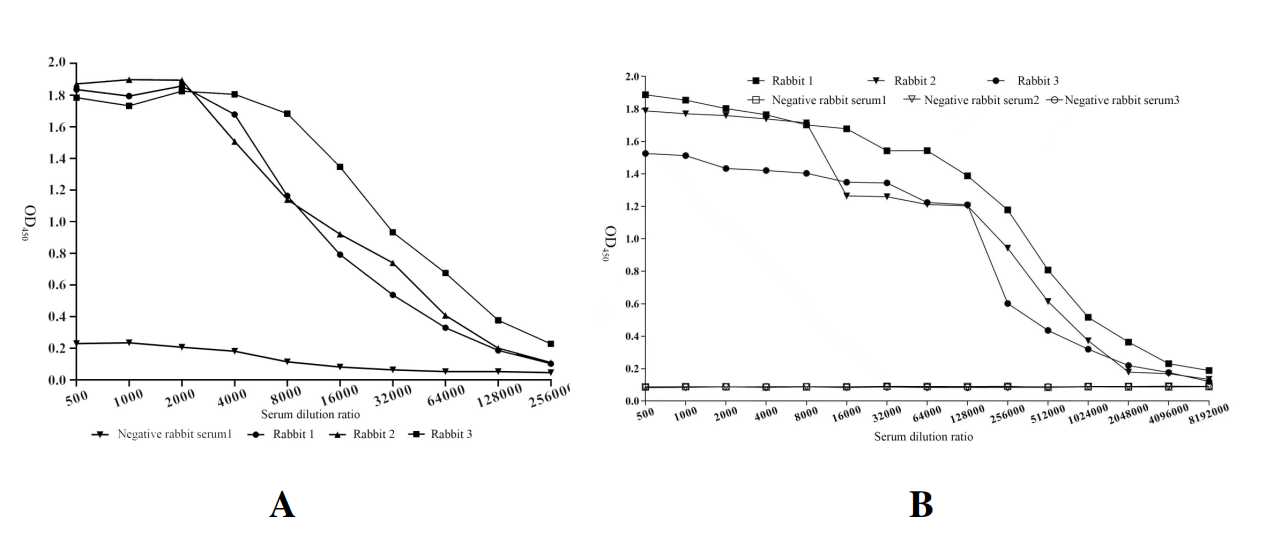


**Fig. S2 Determination of the polyclonal antibody titre of BbPmV-4-CP and BbCV2-CP protein**

1. BbPmV-4-CP.(B) BbCV2-CP.
