# Supplementary material for: Hypovirulence-associated mycovirus epidemics cause pathogenicity degeneration of Beauveria bassiana in the field
Source: Virol J. 2023 Nov 3;20:255. doi: 10.1186/s12985-023-02217-6 (PMC10623766; doi:10.1186/s12985-023-02217-6)
Supplement: Supplementary file 11 — Additional file 11: Fig. S5. Correlation heatmap of strain samples [file 12985_2023_2217_MOESM11_ESM.docx]

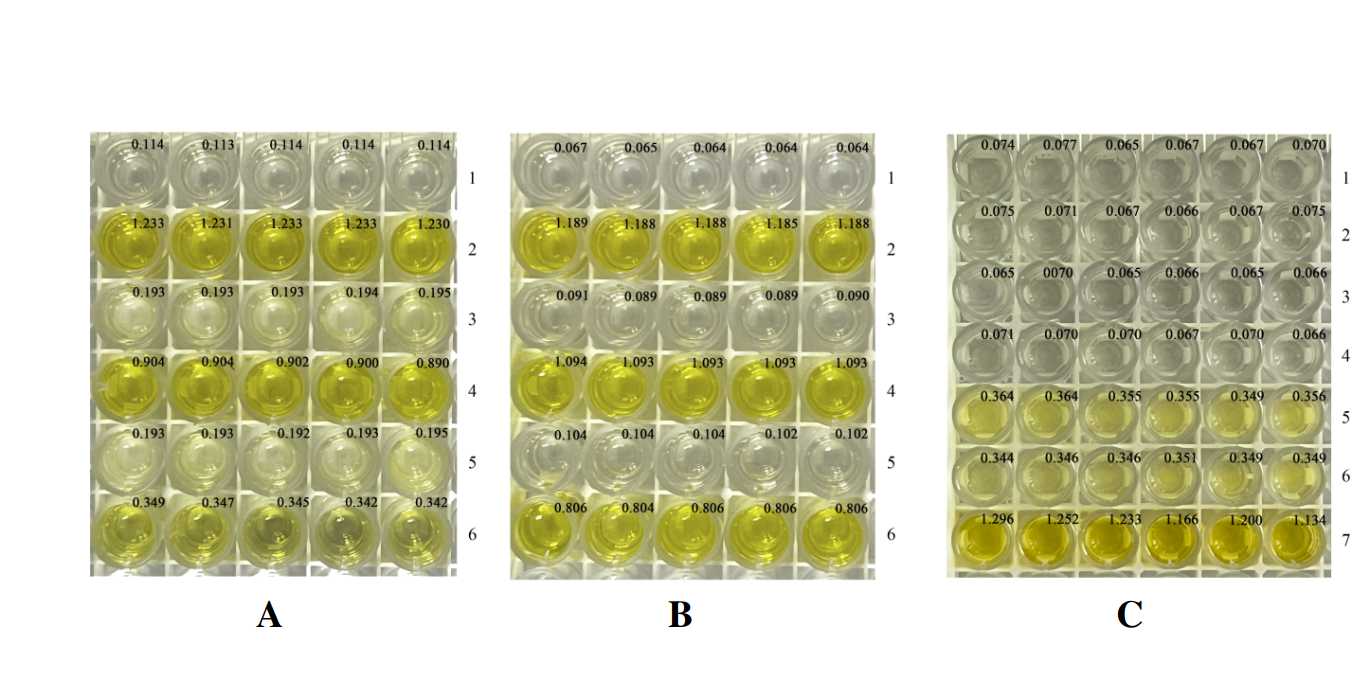


**Fig. S3 Detection of BbCV2 and BbPmV-4 virus in and outside host cells by indirect-ELISA.** (A) Liquid culture medium of *Beauveria bassiana* containing virus BbCV2.1, blank control; 2, positive control (BbCV2-CP); 3, negative control (supernatant of virus-free strains); 4, supernatant of BbCV2 virus-harbouring strains; 5, negative control (pellet of virus-free strains); 6, pellet of BbCV2 virus-harbouring strains; (B) Liquid culture medium of *Beauveria bassiana* containing virus BbPmV-4.1, blank control; 2, positive control (BbPmV-4-CP); 3, negative control (supernatant of virus-free strains); 4, supernatant of BbPmV-4 virus-harbouring strains; 5, negative control (pellet of virus-free strains); 6, pellet of BbPmV-4 virus-harbouring strains. (C) Insect bodies. 1-2, negative control (larvae without *B. bassiana* infection); 3-4, larvae infected by BbOFDH; 5-6, larvae infected by BbOFDHCV; 7, positive control (BbCV2-CP).
