# Supplementary material for: Hypovirulence-associated mycovirus epidemics cause pathogenicity degeneration of Beauveria bassiana in the field
Source: Virol J. 2023 Nov 3;20:255. doi: 10.1186/s12985-023-02217-6 (PMC10623766; doi:10.1186/s12985-023-02217-6)
Supplement: Supplementary file 12 — Additional file 12: Fig. S6. qRT-PCR verification of RNA-Seq gene expression levels [file 12985_2023_2217_MOESM12_ESM.docx]

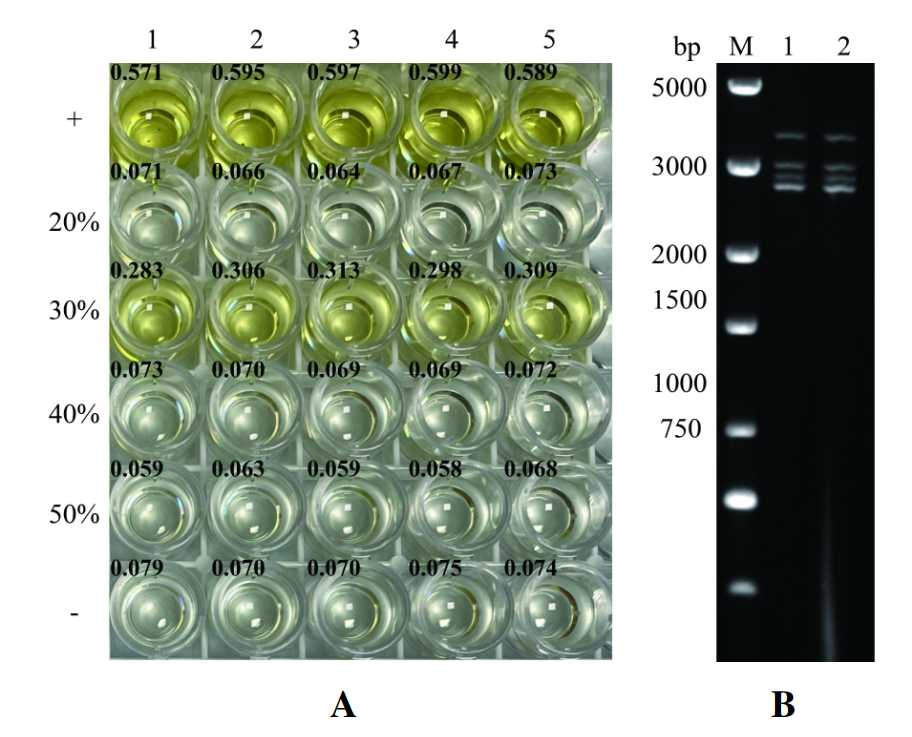


**Fig. S4 Detection of BbCV2 virions by indirect-ELISA and dsRNA extraction.** (A) Detection of virions in different gradients of sucrose by indirect-ELISA.+, positive control (BbCV2-CP); -, blank control; 20%—50%, different gradients of sucrose; (B) DsRNA of virions in 30% sucrose.
